# Preparation and Evaluation of the ZnO NP–Ampicillin/Sulbactam Nanoantibiotic: Optimization of Formulation Variables Using RSM Coupled GA Method and Antibacterial Activities

**DOI:** 10.3390/biom9120764

**Published:** 2019-11-21

**Authors:** Nidhi Sharma, Vineeta Singh, Asheesh Kumar Pandey, Bhartendu Nath Mishra, Maria Kulsoom, Nandita Dasgupta, Saif Khan, Hesham A. El-Enshasy, Shafiul Haque

**Affiliations:** 1 Department of Biotechnology, Institute of Engineering and Technology, Dr. A.P.J. Abdul Kalam Technical University, Lucknow, Sitapur Road, Lucknow-226021, Uttar Pradesh, India; sharmanidhirba@gmail.com (N.S.); vscdri@gmail.com (V.S.); pandeyasish@gmail.com (A.K.P.); profbnmishra@gmail.com (B.N.M.); kulsoom.maria@gmail.com (M.K.); nanditadg254@gmail.com (N.D.); 2 Department of Basic Sciences, College of Dental Sciences, University of Ha’il, Ha’il-2440, Saudi Arabia; saifkhan.bio@gmail.com; 3 Institute of Bioproduct Development (IBD), Universiti Teknologi Malaysia (UTM), Skudai, Johor Bahru 81310, Johor, Malaysia; 4 City of Scientific Research and Technological Applications, New Burg Al Arab, Alexandria 21934, Egypt; 5 Research and Scientific Studies Unit, College of Nursing & Allied Health Sciences, Jazan University, Jazan-45142, Saudi Arabia

**Keywords:** *Klebsiella pneumoniae*, ampicillin/sulbactam, ZnO nanoparticle, response surface methodology, genetic algorithm

## Abstract

Nanoparticles (NPs) possessing antibacterial activity represent an effective way of overcoming bacterial resistance. In the present work, we report a novel formulation of a nanoantibiotic formed using Ampicillin/sulbactam (Ams) and a zinc oxide nanoparticle (ZnO NP). ‘ZnO NP–Ams’ nanoantibiotic formulation is optimized using response surface methodology coupled genetic algorithm approach. The optimized formulation of nanoantibiotic (ZnO NP: 49.9 μg/mL; Ams: 33.6 μg/mL; incubation time: 27 h) demonstrated 15% enhanced activity compared to the unoptimized formulation against *K. pneumoniae*. The reactive oxygen species (ROS) generation was directly proportional to the interaction time of nanoantibiotic and *K. pneumoniae* after the initial lag phase of ~18 h as evident from 2s’-7’-Dichlorodihydrofluorescein diacetate assay. A low minimum inhibitory concentration (6.25 μg/mL) of nanoantibiotic formulation reveals that even a low concentration of nanoantibiotic can prove to be effective against *K. pneumoniae.* The importance of nanoantibiotic formulation is also evident by the fact that the 100 μg/mL of Ams and 25 µg of ZnO NP was required individually to inhibit the growth of *K. pneumonia*, whereas only 6.25 μg/mL of optimized nanoantibiotic formulation (ZnO NP and Ams in the ratio of 49.9: 33.6 in μg/mL and conjugation time of 27 h) was needed for the same.

## 1. Introduction

The emergence of microbial resistance to conventional antibiotics poses a major threat in the field of medicine and makes the bacterial treatment quite difficult [1]. Therefore, there is an urgent need of developing novel therapeutic approaches, such as new drugs and drug targets to treat resistant microbial infections. During the past few decades, nanoparticles (NPs) have been exploited significantly because of their applications in the targeted delivery of therapeutic agents [2], disease diagnosis [3], and minimizing microbial infections [4]. Antimicrobial activities of NPs make it a suitable alternative to these antibiotics that are no longer effective against resistant microbes. 

Nowadays, applications of a combination of NPs with antibiotics, termed as nanoantibiotics, is a novel strategy and therefore gaining research interest as it increases the effectiveness of both the nanoparticle and antibiotic towards multidrug-resistant (MDR) microbes [5]. The conjugation of NPs with small molecules, like drugs showing a synergistic effect, is a promising approach for reducing the emergence of microbial resistance [6]. The application of nanoantibiotics for the treatment of microbial infections not only minimizes the toxicity of both the substances i.e., antibiotics and NPs towards a human cell line, but elevates their antimicrobial properties [7]. Shahverdi et al., (2007) reported an enhancement in the activity of antibiotics (amoxicillin, erythromycin and vancomycin) against *S. aureus* and *Escherichia coli*, when used in a combination with silver nanoparticles [8].

Enormous literature is available on NPs and their applications in the medical field. However, restricted data is available on metal oxide NPs that cover in vivo antimicrobial effectiveness in combination with antibiotics against the MDR pathogens. Among nanoparticles, silver NPs have been studied extensively for antimicrobial activities alone as well as in combination with antibiotics [6]. Silver NPs have been reported to enhance the activity of antibiotics (penicillin G, amoxicillin, erythromycin, clindamycin, and vancomycin) against *Staphylococcus aureus* and *Escherichia coli* [9]. However, the efficacy of other nanomaterials in synergism with antibiotics need to be investigated properly, such as copper oxide (CuO) and zinc oxide (ZnO). Among nanoparticles, NPs of ZnO have effective catalytic efficiency, chemical stability and strong adsorption ability and therefore are gaining more attention from researchers [10,11,12,13]. The antimicrobial potential of ZnO NPs has already been proved and utilized against a variety of bacterial and fungal strains [10,11,12,13,14]. The United States’ Food and Drug Administration (US-FDA) has approved ZnO as a safe material having extensive applications in drug delivery, pharmaceuticals and food supplements etc. [15]

Process optimization is still one of the most critically investigated phenomena that has to be carried out before any large-scale production. There are many techniques, from traditional to advanced, available for process optimization. Traditional optimization methods used for the screening of effective parameters for desirable outcomes are generally based on one-factor-at-a-time (OFAT) approach. OFAT is a time-consuming, cost-effective, labor-intensive methodology and failed to depict the interactions of parameters [16]. These drawbacks of a traditional approach can be overcome by using statistical optimization techniques like response surface methodology (RSM). 

RSM is used for the study of linear, square and interaction effects of the parameters on the process. Statistical optimization techniques are effectively used in diverse fields for various optimization processes, such as metabolite production, metabolite extraction, bacterial cell lysis, etc. [17,18,19] Keeping the potential of statistical optimization techniques in view, researchers have started using various designs of RSM for the formulation optimization of various nanodrug/delivery preparations [20,21,22,23,24,25,26]. However, artificial intelligence (AI)-based optimization techniques alone or in amalgamation with statistical optimization techniques have yet to be used in the formulation optimization of nanodrug preparations. In the present study, we have used a RSM-coupled genetic algorithm (GA) approach for the optimization of formulation variables of the ZnO nanoantibiotic. To the best of our knowledge, this RSM/GA amalgamated optimization approach has not yet utilized in the field of nanoantibiotic formulations.

RSM uses factorial designs, like central composite design (CCD), to optimize any process output. The experimental runs of the CCD act as inputs for RSM in finding the mathematical model, which correlates process parameters and outcome. This mathematical model can be presented in the form of a second-order polynomial equation:

Y = b°+ Σbi Xi + Σbi^2^ Xi^2^ + Σbij Xi Xj (1)

where, Y is the process output, X is the variable, b° is the intercept, bi is the coefficient for linear effect, bi^2^ is the coefficient for quadratic effect and is responsible for curvatures in the model, and bij is the coefficient for the interaction effect. 

A mathematical model of RSM, i.e., Equation (1), serves as a fitness function for the genetic algorithm (GA) to determine the optimum concentrations of the process parameter for maximum process output. The concept of GA method is based on a natural selection process just like biological evolution. On the basis of the rules of selection, crossover and mutation, GA randomly selects the individuals from the current population to act as parents and uses them to produce offspring for the next generation. Over consecutive generations, the population “shifted” toward an optimal solution.

*Klebsiella pneumoniae* is a Gram-negative bacterial strain that is known to cause an array of infections. The emergence of multidrug resistant (MDR) strains of *K. pneumonia* has narrowed down the current therapeutic interventions. Therefore, this study was performed to develop an effective way of reutilizing the presently available therapeutic molecule in a more efficient manner by exploiting the potential of ZnO NP against *K. pneumoniae*. In the present study, antibacterial efficacy of different antibiotics was evaluated against different microorganisms using the agar well diffusion method. The most resistant antibiotic found, i.e., Ampicillin/Sulbactum (Ams), was conjugated with ZnO NPs. To the best of our knowledge, for the first time, we have prepared and optimized the formulation of ZnO NP–Ams nanoantibiotic using traditional -and currently used RSM-coupled GA method followed by its evaluation and testing of antibacterial activities. The graphical abstract appended with this manuscript shows the representation of the complete process of preparation, optimization and evaluation of ZnO NP–Ams nanoantibiotic effective against *K. pneumoniae.*

## 2. Results 

### 2.1. Selection of Drug for Nanoantibiotic Formulation

The results regarding the antibiotic resistance profile of six selected bacterial strains against 17 antibiotics are summarized in Table 1. ‘Ampicillin/Sulbactam’ (Ams) showed maximum resistance against five bacterial strains (*Streptococcus aureus* MTCC 902*, Escherichia coli* MTCC 1304*, Klebsiella pneumoniae* MTCC 3384, *Pseudomonas aeruginosa* MTCC 741 and *Salmonella typhi* MTCC 537) followed by ampicillin, amoxyclav, ceftazidime and penicillin G. Ams showing resistance in maximum microbial cases was selected for further study.

### 2.2. Minimum Inhibitory Concentration (MIC) of Ams against Resistant Bacterial Strains 

Increased antibiotics doses are sometimes able to inhibit the resistant pathogens. To investigate this, enhancement in the inhibitory concentration of Ams against resistant pathogens, MIC was evaluated against *E. coli, K. pneumoniae*, *P. aeruginosa*, *S. typhi* and *S. aureus*. The results suggest that the tested bacterial strains resistant at lower (10 μg) concentration of Ams (Table 1), becomes sensitive at a higher Ams concentration (Table 2). One-hundred μg/mL of Ams was required to inhibit the growth of *P. aeruginosa* and *K. pneumoniae,* whereas 50 μg/mL was sufficient to inhibit the growth of *S. typhi, S. aureus,* and *E. coli*. 

### 2.3. Antibacterial Activity of ZnO NP

Antibacterial activity of ZnO NPs was observed at four different concentrations (µg/mL) i.e. 25, 50, 100, and 200, respectively. The results suggest that ZnO nanoparticles possess inhibitory activity against all the tested bacterial strains (Table 3). With the increase in the concentration of ZnO NP, enhancement in its inhibitory activity against the examined bacterial strains was observed. ZnO NP showed maximum activity against *K. pneumoniae* followed by *S. typhi*, *E. coli*, *S. aureus*, and *P. aeruginosa.* Based on the promising results of ZnO NP against *K. pneumoniae,* this strain was selected for further experimental study.

### 2.4. ‘ZnO NP–Ams’ Nanoantibiotics—Formulation and Optimization Employing Statistical Design

The responses of the concentration of ZnO NP, Ams antibiotics, and their conjugation time on the effect of ZnO NP–Ams nanoantibiotic observed by executing the RSM experiments are summarized in Table 4. 

The results of the regression coefficient of linear, interaction and quadratic terms of the above variables are listed in Table 5. Linear value of ZnO NP concentration (Var1: *p* = 0.13150) and Ams concentration (Var2: *p* = 0.07796) was found to be insignificant, whereas their quadratic values were found to be significant (Var1: *p* = 0.01155; Var2: *p* = 0.00492). However, the conjugation time (Var3) was found to be significant in term of linear (Var3: *p* = 0.00196) as well as quadratic (Var3: *p* = 0.00045). The interaction of nanoparticle concentration (Var1) and Ams concentration (Var2) was found to be significant but the interaction of the conjugation time (Var3) with ZnO NP concentration (Var1) and Ams concentration (Var2) was found to be insignificant.

#### 2.4.1. ANOVA Analysis

Regression coefficients were further validated by the analysis of variances (ANOVA). The results confirmed the adequacy of the developed response surface model (Table 6). 

The low probability value of the Fisher *F*-test (*F* = 6.70, *p* = 0. 0031) indicates the statistical significance of the regression model. The high value of the correlation coefficient (R = 0.926) of model explains the significant interactions among the independent variables. The determination coefficient (R^2^ = 0.857) indicates the goodness-of-fit of the model and suggests that the generated second-order polynomial model was able to interpret 85.7% of the input data. The adjusted determination coefficient (adj. R^2^ = 0.73001) of the model further indicated its high significance. 

The model generated through Statistica version 10 is expressed in the form of second-order polynomial equation shows a relationship between the logarithmic values of dependent (nanoantibiotic) and independent variables (ZnO NP, antibiotics and conjugation time).

Y = 15.00335 + 0.114000000(Var1) − 0.00121818(Var1)^2^ + 0.170681818(Var2) − 0.00221591(Var2)^2^ + 0.596496212(Var3) − 0.00875947(Var3)^2^ + 0.002000000(Var1)(Var2) − 0.000833(Var1)(Var3) − 0.00312500(Var2)(Var3) (2)

where, Y is a response, i.e., zone of inhibition (mm). Var1 = concentration of ZnO nanoparticles, Var2 = concentration of Ams antibiotics and Var3 = interaction time between nanoparticles and antibiotics.

#### 2.4.2. Contour Plots

**Figure 1:**
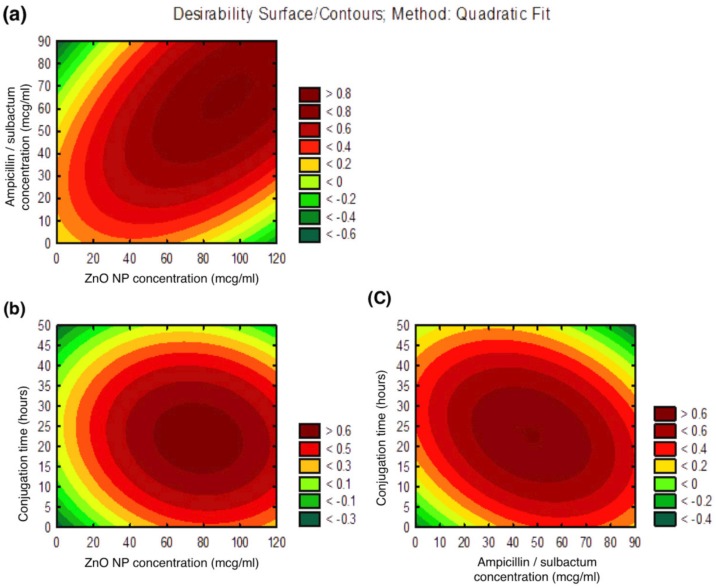
Response Surface Contour plots: (**a**) Contour plot of ZnO NP and Ampicillin/sulbactum concentration; (**b**) Contour plot of ZnO NP and conjugation time; (**c**) Contour plot of Ampicillin/sulbactum concentration and conjugation time.

The three-dimensional contour plots (Figure 1a–c) obtained through Statistica V10 explain the main and the interaction effects of two variables. These plots represent the effect of two variables on the output at a time while maintaining the third variable fixed at zero levels (coded units). The first graph (Figure 1a) between ZnO NP and Ams concentration, shows the requirements of increase in the values of both the variables for higher antibacterial activity of the formulated ZnO NP–Ams nanoantibiotic. The second graph (Figure 1b) between ZnO NP and conjugation time, suggests the higher value of ZnO NP and lower value of conjugation time are required to maximize the nanoantibiotic’s effect. The third graph (Figure 1c) between Ams and conjugation time (Var3) shows higher value of Ams and lower value of conjugation time favors high antibacterial activity of the formulated nanoantibiotic. 

### 2.5. Genetic Algorithm-Based Optimization 

**Figure 2:**
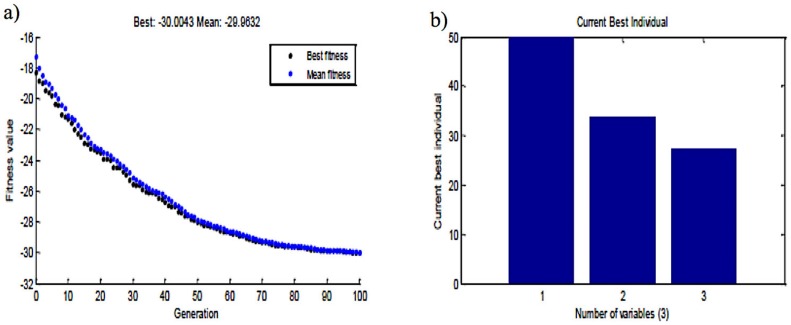
Genetic algorithm showing generations till the optimum results obtained, the optimum conditions of the nanoantibiotic components: (**a**) Current generation vs. fitness values; (**b**) Current best individual vs. number of variables.

The genetic algorithm (GA) was employed using MATLAB to optimize the second-order polynomial model developed through RSM. The genetic algorithm depicted the optimum concentration of ZnO NP concentration (Var1), Ams concentration (Var2) and conjugation time (Var3) as 49.9 μg/mL, 33.6 μg/mL and 27 h, respectively, at which, antibacterial activity in terms of zone of inhibition (zoi) is 30 mm (Figure 2a,b).

The results suggest that nanoantibiotic formulated through optimized parameters demonstrates 15% activity enhancement compared with the unoptimized parameter. The predicted yield was experimentally verified with the optimized concentrations in a basal medium and the zone of inhibition was around 29 mm (i.e., very close to the predicted one). The zone of inhibition at the center points of CCD was observed as 26 mm. The comparison of CCD results (center point: 26 mm) with the optimized one (29 mm) further suggests that the GA-optimized concentration was better than the CCD designed concentration.

### 2.6. ROS Estimation

**Figure 3:**
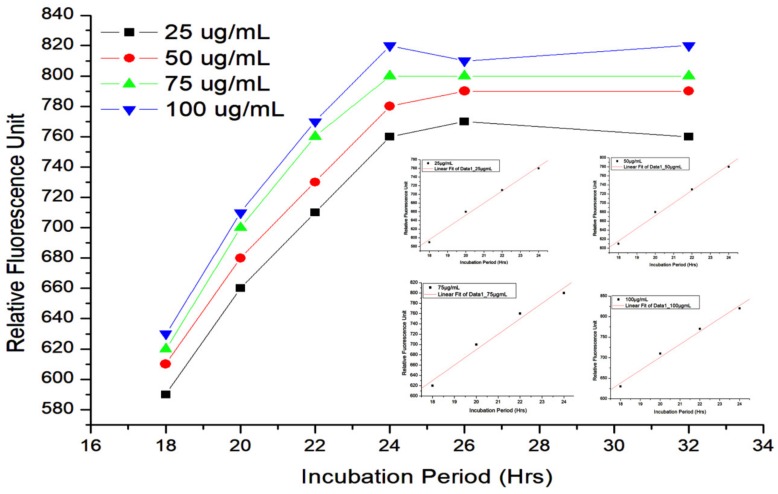
Cell-based DCFH-DA assay graph of ROS estimation. *Note:* Each bar represents the mean of two technical duplicates of an independent experiment and the corresponding standard deviations.

The mode of action of the optimized (formulated) nanoantibiotic (consisted of 55 µg/mL concentration of ZnO NPs + 45 µg/mL concentration of ampicillin/sulbactam) in Ams-resistant *K. pneumoniae* cells was studied by ROS estimation employing the DCFH-DA assay. The results of DCFH-DA assay have been summarized in Figure 3, which shows the fluorescence intensity measured at different times points using different concentrations of formulated ZnO NP–Ams nanoantibiotic. 

DCFH-DA assay revealed that ROS generation is directly proportional to the interaction time of ZnO NP–Ams and *K. pneumoniae* after the initial lag phase of ~18 h. In other words, a time dependent increment in the fluorescence level was noticed after the initial lag of some hours. Besides, it is also clear from the assay results that with the increase in the interaction time, relative fluorescence unit (RFU) also increases. 

### 2.7. Determination of MIC of Optimized ZnO NP-Ampicillin/Sulbactam Nanoantibiotic 

MIC experiment was performed to determine the minimum concentration of ZnO NP–Ams nanoantibiotic, required to inhibit the growth of *K. pneumoniae*. The MIC of optimized nanoantibiotic to inhibit the growth of *K. pneumoniae*, which consists of the combination of ZnO NP and Ams in the ratio of 49.9: 33.6 in μg/mL and conjugation time of 27 h was found to be 6.25 μg/mL.

### 2.8. Scanning Electron Microscopy

**Figure 4:**
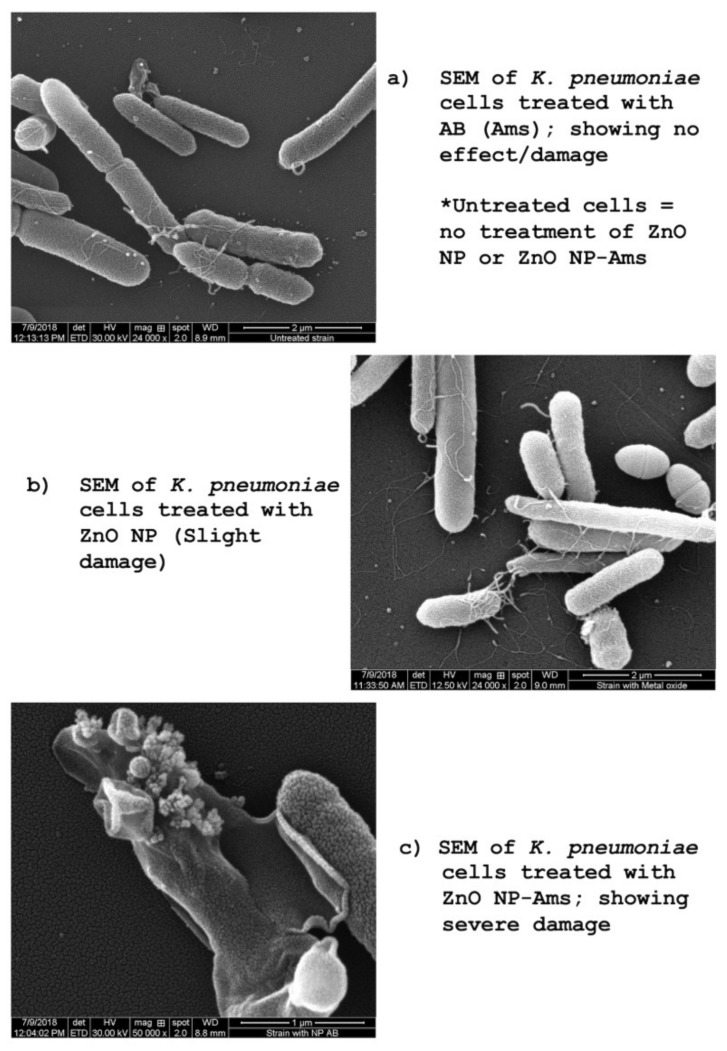
Scanning electron microscopy of treated *K. pneumoniae* cells: (**a**) SEM of *K. pneumoniae* cells treated with Ams (AB); showing no effect/damage; (**b**) SEM of *K. pneumoniae* cells treated with ZnO NP; slight damage; (**c**) SEM of *K. pneumoniae* cells treated with ZnO NP–Ams (AB); showing severe damage. ***Note:*** *Untreated strain = no treatment of Ams, ZnO NP, or ZnO NP–Ams.

The activity of the optimized (formulated) ZnO NP–Ams nanoantibiotic was further corroborated by scanning electron microscopy (SEM) as shown in Figure 4a–c. SEM images of ZnO NP, Ams, and ZnO NP–Ams nanoantibiotic treated *K. pneumoniae* revealed that optimized formulated nanoantibiotic caused severe damage to the microbial cell compared to ZnO NP or Ams alone and endorsed its efficacy.

## 3. Discussion

Ampicillin, a broad spectrum antibiotic of the β-lactam class is well known for its applications for treating infections, such as respiratory tract infections, urinary tract infections, meningitis, salmonellosis and endocarditis caused by Gram-positive and -negative bacteria. The antibacterial activity of ampicillin is demonstrated by the inactivation of D, D-transpeptidases enzyme, responsible for the cross-linkage of peptidoglycan moieties during the synthesis of the cell wall [27]. However, some of the bacteria, with time, was used to develop resistance to ampicillin by producing a β-lactamase/penicillinase enzyme like TEM-1, TEM-2 or SHV-1 that attacks the β-lactam ring of antibiotics [28]. To overcome this problem, β-lactam antibiotics are now given with β-lactamase inhibitors, such as sulbactam, with clavulanic acid having the ability to inhibit the β-lactamase enzymes produced by bacteria [29]. With the emergence of extended spectrum β-lactamase (ESBL), a mutant of the β-lactamase enzyme, the incidences of resistance against this combination have also been observed in hospitals [30]. *Pseudomonas aeruginosa, Klebsiella pneumoniae*, *Salmonella typhi* and *Escherichia coli* are some of the ESBL-producing strains that render them resistant to various antibiotics [31,32,33,34]. The application of Ams for the treatment of infections is now becoming ineffective. Since *K. pneumoniae* is an ESBL-producing strain that causes a range of minor to life-threatening nosocomial infections, it was considered for this study [32]. 

During the experiments, *K. pneumoniae* was found resistant to Ams at lower (10–50 µg) concentrations. At higher concentrations (≥100 µg/mL), Ams is effective, however, it may lead to severe side effects. Therefore, an alternate approach is the need of an hour. The use of nanoparticles is one of the best possible solutions to meet such a need. Hence, we studied the activities of ZnO NP of dimension 25 nm in combination with Ams for the formulation of a potential nanoantibiotic. Earlier studies proved that with the decrease in size, the efficiency of nanoparticles increases. Nanoparticles show unique properties that are significantly different from their bulk counterparts. Due to the decrease in size, they can be engineered for different end applications [35]. The properties of metal oxides, such as zinc oxide (ZnO), greatly depend upon their size, shape, composition and morphology. A reduction in size, allows nanoparticles to interact more efficiently with cellular biomolecules and facilitate easier penetration into the cell. A large surface to volume ratio of ZnO nanoparticles increases the surface reactivity, as a large number of free electrons are available at the surface [36].

The combination of ZnO NP–Ams was found reasonably effective against Ams-resistant *K. pneumoniae* even at a lower concentration. Further, we optimized the formulation of this combination using experimental designs. The statistical/AI approaches like RSM, ANN and GA are some popular techniques used in the optimization of various parameters like metabolite production, extraction condition etc. [16,17,37]. Response surface methodology is the most commonly used statistical technique used for depicting the nature of the response within the framed design space [17], whereas the genetic algorithm mimics the biological mutation process; hence it is based upon the biological principle of “survival of the fittest”. The theory similar to natural selection (biological process of evolution) plays a significant role in the execution of tool [16].

In the present work, we applied RSM and GA in combination to optimize three parameters i.e., NP concentration, Ams concentration and their conjugation time for the formulation of a nanoantibiotic. The conjugation time plays an important role in the formation of nanoantibiotic by facilitating the maximum uptake of either the antibiotic or nanoparticle at the surface of each other and may vary from 12 to 48 h, depending on the nature of nanoparticles [38,39]. The application of the RSM and GA methodology has significantly improved (nearly 15%) the antibacterial activity of the ZnO NP–Ams nanoantibiotic formulation compared with the unoptimized conditions. The approach presented here is significantly simple in nature and can be extended for modeling and optimization of other similar processes. 

An explanation for the increased activity of Ams in the presence of ZnO NP in *K. pneumoniae* is based on the fact that the ZnO NP generates reactive oxygen species (H_2_O_2_ and OH) and also possesses the ability to induce faster electron transfer kinetics at the active site of the enzyme [27,40]. NPs are well known to interact with the basic components of bacterial cells, which leads to oxidative stress, changes in membrane permeability, heterogeneous alterations, enzyme inhibition, protein deactivation, changes in gene expression, etc. [41]. Arakha et al., (2015) hypothesized that the neutralization of bacterial surface potential leads into electron–hole pair generation in proximity, which ultimately enhances ROS production [42]. In this study, the change in ROS production due to the addition of ZnO NPs has been evaluated using the fluorescence dye DCFH-DA [43]. In literature, DCFH-DA used as ROS indicator, is basically a peroxynitrite indicator, which is capable of detecting hydrogen peroxide and nitric oxide [44]. The interaction studies of the nanoantibiotic and the cells of *K. pneumoniae* at different interaction times (1 to 34 h; measured at the interval of every 2 h) through DCFH-DA suggested that nanoantibiotic treated microbial cells exposed for a longer time period leads to more ROS generation as evident by the increased fluorescence. This in turn increases the activity of the nanoantibiotic. Also, during the experiments, minor ROS generation was observed even in the absence of ZnO NPs, i.e., control culture. Some ROS scavenging enzymes are present in bacteria, which counteract the ROS produced under non-stress condition [29]. However, in the presence of NPs, this ROS production is comparatively high. Tiwari et al., (2018) reported a fourfold enhancement in the production of ROS in the cells of ZnO NP-treated, carbapenem-resistant *Acinetobacter baumannii* compared to the untreated ones [45]. Yi et al., (2019) have evaluated the production of ROS by using 10 mg of ZnO NP alone in the dark and the absorbance at a wavelength of 470 nm was observed to be 0.05. On the other hand, we evaluated ROS generation in µg levels of a nanoantibiotic (ZnO NP–Ams) treatment and the fluorescence intensity value ranged between 500–800 [46]. Thus, the ROS generated by ZnO nanoparticle is negligible compared to ROS generated by the bacteria.

The chemical interaction between Ams and ZnO NP is still unexplored. However, it can be hypothesized that the interaction of the antibiotic with the nanoparticle (ZnO NP) is reversible in nature, as the surface of ZnO NP and other metal-oxide NPs is rich in surface bound hydroxyl groups, which increase the Zeta (ζ) potential of ZnO NP [47]. This facilitates a reversible ionic interaction with the surface-bound molecules, which may also undergo reversible hydrogen bonding [47]. The bound antibiotic in a reversible interaction can only be released and interact with its target. In contrast, irreversible interaction (covalent bond formation) between the adsorbent (ZnO NP) and adsorbate (antibiotic) may result in permanent linkage of the antibiotic and ZnO NP, which ultimately render the antibiotic inactive.

Hence, at any instance, the interaction of antibiotic the nanoparticle (within the medium) can be represented by an equilibrium (K).

A (antibiotic) + N (free nanoparticle) ↔ NA (antibiotic nanoparticle complex)

K = [NA]/[A]·[N](3)

where, K is an Equilibrium constant and depends upon the type and properties of the nanoparticle and antibiotic under consideration.

From the above equilibrium, it is evident that the free nanoparticles are always available and remain in a dynamic equilibrium with the coated ones. These free NPs are responsible for interacting with the microbial cell wall through several reported mechanisms including ROS generation [48,49,50,51,52,53]. This further facilitates the interaction of the positively charged ZnO NP with the negatively charged molecules on the cell surface and providing channels for easy delivery of the antibiotic [54]. 

Basically, lipopolysaccharides present in the cell wall of Gram-negative bacteria, provides a negatively charged region on the cell surface that attracts positively charged ZnO NPs [31]. Hence, speculations can be made that ZnO NP firstly comes in contact with the bacterial cells by various forces such as electrostatic attraction, van der Waals forces, receptor–ligand and hydrophobic interactions that neutralizes the surface charge (potential) of the bacterial cell, which leads to the enhanced production of ROS [41]. The produced ROS may create some electron holes on the surface of bacterial cells, which facilitate the entry of ZnO NP and Ams both. The free radicals generated by ZnO NP may oxidize the ESBL enzyme of *K. pneumoniae*, which is responsible for the resistivity towards Ams [55,56]. ZnO NP hampers the ESBL enzyme present in the cytoplasm and also changes the permeability of the cell wall of bacteria. This in turn might enhance the entry of Ams into *K. pneumoniae* cells, and allow for the antibiotic to perform its own mechanism of action i.e., inhibition of cell wall synthesis by irreversibly binding to the active site of transpeptidase enzyme [57]. Hence, both ZnO NP and Ams works synergistically against the Ams-resistant *K. pneumoniae*. 

Earlier, Pati et al., (2014) reported the increased killing of BCG using a combination of rifampicin and ZnO-NPs [58]. For the mechanism of antibacterial activity of the formulated nanoantibiotics, Pati et al. (2014) hypothesized that ZnO-NPs may facilitate the transport of rifampicin inside the mycobacterial cells by altering cell membrane permeability and thereby killing of mycobacteria by rifampicin’s mode of action of RNA synthesis inhibition [58]. The membrane integrity of *K. pneumoniae* in the presence of a nanoantibiotic was studied by cell wall disruption and identified by the analysis of scanning electron microscopy technique. SEM analysis assists in the prediction that ZnO NPs that interact with the microbial surface may lead to the disruption of the cell membrane.

The material safety data sheet in accordance with Occupational Safety and Health Administration (OSHA) and American National Standards Institute (AN.S.I) says that ZnO NP LD_50_ is 8437 mg/kg in rats, i.e., 8437 mg of ZnO NP per kg weight of NPs causes death in 50% of rats, which reflects the toxicity of ZnO NPs at higher concentrations. The present study demonstrates that 49.9 μg/mL of ZnO NPs and 33.6 μg/mL concentration of ampicillin/sulbactam were used to formulate the nanoantibiotic. The formulated nanoantibiotic was subjected to MIC analysis and it was found that 6.25 μg/mL of this formulation, i.e., ZnO NP–Ams nanoantibiotic, was sufficient to inhibit the growth of *K. pneumoniae*. Hence, it can be assumed that the toxicity profile of this combination of nanoantibiotic will be very low in humans. However, in vitro and in vivo studies are still required to justify and substantiate this assumption. 

## 4. Conclusions

In the recent past, a plethora of efforts have been made to overcome the emerging problem of antibiotic resistance against various bacterial diseases, and advances in the field of nanobiotechnology may offer a great opportunity for research in this field. Thus, studies based on the combination of antibiotic agents and nanomaterials are of great promise. In the present study, for the first time, we report a significant improvement and reversal of antibacterial activity of Ams against Ams-resistant *K. pneumoniae* in the presence of ZnO NPs (25 nm in size) using the RSM-coupled GA formulation optimization technique. Due to the potential synergistic effect of ZnO NP with Ams, ZnO NP may be considered as a valuable adjuvant in the case of Ams-resistant *K. pneumoniae*. The application of RSM-GA optimization has significantly enhanced the antibacterial activity of the formulated ZnO NP–Ams nanoantibiotic. The antibacterial activity of the formulated and optimized ZnO NP–Ams was increased by 15% when compared with the unoptimized combination. However, further studies related to pharmacokinetics, tissue distribution and excretion of the proposed nanoantibiotic, along with the elucidation of their mechanistic action, are warranted for the development of safe, efficient, cost-effective, and targeted therapy. Additionally, future in silico studies are required to study the chemical interactions between an antibiotic moiety–ZnO NP combination.

## 5. Materials and Methods

### 5.1. Bacterial Strains, Culture Conditions and Antibiotics

Six bacterial strains, *Bacillus pumilus* MTCC 1607, *Streptococcus aureus* MTCC 902, *Escherichia coli* MTCC 1304, *Klebsiella pneumoniae* MTCC 3384, *Pseudomonas aeruginosa* MTCC 741 and *Salmonella typhi* MTCC 537, were grown at 160 rpm in nutrient broth (NB) at 32 °C. Seventeen antibiotics discs named Amikacin (Ak) 30 µg, Ampicillin (A) 10 µg, Ampicillin/Sulbactam (Ams) 10/10 µg, Amoxyclav (Ac) 30 µg, Ceftazidime (Ca) 30 µg, Cephotaxime (Ce) 30 µg, Ciprofloxacin (Cf) 5 µg, Clindamycin (Cd) 2 µg, Co- Trimoxazole (Co) 25 µg, Erythromycin (E) 15 µg, Gentamycin (G) 10 µg, Nalidixic acid (Na) 30 µg, Netillin (Nt) 30 µg, Nitrofurantoin (Nf) 300 µg, Penicillin G (P) 10 units, Tobramycin (Tb) 10 µg and Vancomycin (Va) 3 µg, were procured from HiMedia Laboratories Pvt. Ltd., India.

### 5.2. Antibiotic Resistance Profile of Bacterial Strains

The antibiotic resistance profile of the microbes was examined using a modified Kirby–Bauer disk Diffusion method [59]. A bacterial lawn was prepared on NA plates using the working solution of the bacterial suspension containing 10^6^ CFU/mL (according to McFarland standards). The disks of seventeen antibiotics were placed on the plates; the plates were incubated overnight at 32 ± 2 °C and the zone of inhibition (mm) was measured.

### 5.3. Minimum Inhibitory Concentration of Antibiotic Against Different Bacterial Strains 

The minimum inhibitory concentration (MIC) of antibiotics against the microbial strains (as mentioned in Section 2.1) was determined by the broth dilution method using NCCLS protocol [60]. The stock concentration of antibiotic (1 mg/mL) was prepared in Dimethyl sulfoxide (DMSO) for the study. One-hundred µg/mL antibiotic concentration was taken as the initial concentration and it was further diluted up to 0.3906 µg/mL. The inoculated sets were incubated at 30 ± 2 °C for 18–24 h. The lowest concentration that inhibits the bacterial growth was recorded as the MIC value. All the experiments were performed in triplicate.

### 5.4. Activity of ZnO NP against Different Bacterial Strains

Chemically synthesized ZnO NP of 25 nm in size was purchased from Reinste Nanoventures Private Limited, New Delhi, India. ZnO NPs used in the present study were synthesized by using the method of Moballegh et al., (2007) [61]. ZnO NPs synthesized by this method are well reported to possess overall positive surface charge [61]. ZnO NPs are known to have high isoelectric point, hence in aqueous medium at lower and near physiological pH the overall charge on the surface of ZnO NP is positive [47]. It has solubility in the ultrapure water. ZnO NP suspension of 1 mg/mL concentration was prepared in MilliQ water followed by continuous stirring for proper mixing. The antimicrobial activity of the NP was determined by using the agar disc diffusion method. Discs with different concentrations (200, 100, 50 and 25 µg) were prepared from the stock suspensions and used for examining the zone of inhibition.

### 5.5. Formulation and Optimization of Nanoantibiotics

The nanoantibiotic (ZnO NP–Ams) was formulated according to the method given by Hussein-Al-Ali et al. (2014) with minor modifications [38]. Ams (1 mg/mL) solution was made in ultra-pure MilliQ water. Likewise, the colloidal solution of ZnO NP (1 mg/mL) was also prepared. The nanoantibiotic (ZnO NP–Ams) was prepared by mixing both the solutions. The above solution mix was stirred at 120 rpm at room temperature for 24 h to facilitate the formulation of nanoantibiotic. The formulation and optimization studies of nanoantibiotic were performed in two stages. In the first stage, the components having a significant effect on the formulation were identified by using one-factor-at-a-time (OFAT) experiments. In the second stage, the optimum values of these components for the formulation of nanoantibiotic were determined by the Central Composite Design (CCD) of Response Surface Methodology (RSM) and Genetic Algorithm (GA).

Table 4 shows the CCD design of three test variables (ZnO NP concentration, Ams concentration, and conjugation time of ZnO NP–Ams in coded and uncoded units) that affects the formulation of nanoantibiotic. The coded units are the values that are mentioned in the design (−2, −1, 0, +1, +2), whereas the uncoded units are the values that were actually taken in the experiments. All the three test variables were varied at five levels (−2, −1, 0, +1, +2). Each row of CCD represents the separate experimental runs. The statistical software Statistica 10.0 was used to perform regression and graphical analysis of the results obtained from CCD. A second-order polynomial response equation comprising of linear, quadratic and interaction terms showing an empirical relationship between the nanoantibiotic effect and the test variables was obtained on applying the RSM.

Y = b° + b1·X1 + b2·X2 + b3·X3 + b4·X1^2^ + b5·X2^2^ + b6·X3^2^ + b7·X1X2 + b8·X1X3 + b9·X2X3 (4)

where Y is the zone of inhibition in mm, b is the coefficient term, X1 = concentration of ZnO nanoparticles, X2 = concentration of Ams antibiotics and X3 = interaction time between nanoparticle and antibiotic.

### 5.6. GA Optimization

The genetic algorithm approach was also used to optimize the formulation and production of the nanoantibiotic. Genetic algorithm program of MATLAB suite/tool was used for the optimization of the model generated via RSM. The input parameters which were considered in ‘ga’ function were as follows-Population Type: ’double Vector’; Pop Init Range: [2×1 double]; population Size: 200; elite count: 2; crossover fraction: 1; migration direction: ’forward’; migration interval: 20; migration fraction: 0.2000; generations: 100; time limit: Inf; fitness limit: -Inf; stall gen limit: 50; stall time limit: 20; initial population: []; initial scores: []; plot interval:1; creation fcn: @gacreationuniform; fitness scaling fcn: @fitscalingrank; selection fcn: @selectionstochunif; Crossover Fcn: @crossoverscattered; mutation fcn: {[1×1 function_handle] [1] [1]}; hybrid fcn: []; display: ’off’; plot fcns: {[1×1 function_handle] [1×1 function_handle]}; output fcns: []; vectorized: ’off’.

The optimized results were also validated in the lab by measuring the zone of inhibition (mm) of the optimized ZnO NP–Ams nanoantibiotic against *K. pneumoniae.* Besides, the minimum inhibitory concentration of the optimized nanoantibiotic against *K. pneumoniae* was also determined by the broth dilution method as established by NCCLS guidelines.

### 5.7. Estimation of Reactive Oxygen Species

ROS was measured by using an oxidation-sensitive fluorescent probe 2,7-dichloro dihydro fluorescein diacetate (DCFH-DA) [62]. The bacterial cells were harvested from the overnight grown culture by centrifugation at 3000 rpm for 5 min. The cells were washed twice and resuspended in NB medium. The cells were exposed to DCFH-DA (working concentration 10 μM) for 30 min at 32 °C in the dark. Further, the cells were diluted to a concentration of 5.0 × 10^8^ cell/mL. A volume of 200 μL of this suspension was kept in each well of 96 well plate so that each well contained nearly 1.0 × 10^8^ cells/well. The ZnO NP: Ams nanoantibiotic was formulated by using the optimized concentration ratio. The cells were treated with different concentrations of optimized nanoantibiotic formulations (10 to 100 μg/mL) for different incubation (1 to 34 h, with a gap of 2 h each) periods at 32 °C. After the incubation, the fluorescence intensity of DCFH-DA was observed for ROS production by fluorescence spectrophotometer at an excitation wavelength of 488 nm and an emission wavelength of 535 nm.

### 5.8. Scanning Electron Microscopic Examinations

Scanning electron microscopy (SEM) was performed for *K. pneumoniae* cells treated at IC_50_ value concentration of ZnO NP and Ams, separately, and optimized formulation of ZnO NP–Ams nanoantibiotic for determining the morphological alterations, if any. The treated cells of *K. pneumoniae* were fixed with 2.5% glutaraldehyde in a phosphate buffer having pH 7.2. The samples were post-fixed in 1% osmium tetroxide, afterwards dehydrated through an ascending ethanol series, critical point dried and coated with Au–Pd (80:20) using a Polaron E5000 sputter coater, keeping the cut surface of the beads facing upwards on the stubs. The samples were checked at an accelerating voltage of 25 kV in FEI Quanta 250 using a SE detector.

## Figures and Tables

**Table 1:** Resistance profile of bacterial strains against different antibiotics.

S. No.	Antibiotic (Concentration)	Zone of Inhibition (mm)					
		*S. a*	*E. c*	*B. p*	*S. t*	*K. p*	*P. a*
1.	Amikacin (Ak^30^)	30	22	25	26	29	19
2.	Ampicillin (A^10^)	R	10	19	R	R	R
3.	Ampicillin/Sulbactam (As^10^)	R	R	11	R	R	R
4.	Amoxyclav (Ac^30^)	R	21	24	R	R	R
5.	Ceftazidime (Ca^30^)	30	R	R	R	13	R
6.	Cephotaxime (Ce^30^)	R	R	18	11	R	11
7.	Ciprofloxacin (Cf^5^)	19	30	24	25	31	29
8.	Clindamycin (Cd^2^)	35	R	21	20	33	10
9.	Co-Trimoxazole (Co^25^)	R	23	33	24	R	29
10.	Erythromycin (E^15^)	28	10	R	10	31	20
11.	Gentamycin (G^10^)	26	23	22	18	24	11
12.	Nalidixic acid (Na^30^)	11	R	26	18	25	30
13.	Netillin (Nt^30^)	12	13	15	12	17	11
14.	Nitrofurantoin (Nf^300^)	R	20	23	21	18	10
15.	Penicillin G (P^10^)	R	20	26	R	R	R
16.	Tobramycin (Tb^10^)	15	13	20	15	20	11
17.	Vancomycin (Va^30^)	R	23	21	16	19	16

Note: S. a (*Staphylococcus aureus* MTCC 902), E. c (*Escherichia coli* MTCC 1304), B. p (*Bacillus pumilus*), S. t (*Salmonella typhi* MTCC 537), K. p (*Klebsiella pneumoniae* MTCC 3384), P. a (*Pseudomonas aeruginosa* MTCC 741); R: Resistance.

**Table 2:** Minimum inhibitory concentration (MIC) of ampicillin/sulbactam (Ams).

S. No.	Bacterial Strains	MIC (μg/mL)
1.	*Escherichia coli* MTCC 1304	50
2.	*Klebsiella pneumoniae* MTCC 3384	100
3.	*Pseudomonas aeruginosa* MTCC 741	100
4.	*Salmonella typhi* MTCC 537	50
5.	*Staphylococcus aureus* MTCC 902	50

**Table 3:** Activity of zinc oxide nanoparticle against different bacterial strains.

S. No.	Bacterial Strains	Zone of Inhibition (in mm) at Different Concentration (µg)			
		25	50	100	200
1.	*Escherichia coli* MTCC 1304	9	10	11	13
2.	*Klebsiella pneumoniae* MTCC 3384	19	20	22	25
3.	*Pseudomonas aeruginosa* MTCC 741	5	6	8	10
4.	*Salmonella typhi* MTCC 537	12	14	18	20
5.	*Staphylococcus aureus* MTCC 902	7	10	14	16

**Table 4:** CCD for formulation and optimization of ‘ZnO NP–Ams’ nanoantibiotic.

Runs	X_1_ Coded Uncoded		X_1_ Coded Uncoded		X_1_ Coded Uncoded		ZOI (mm) Experimental Predicted Residual		
1.	+1	80	+1	65	+1	36	26	24.56	1.44
2.	+1	80	−1	25	−1	12	25	23.06	1.94
3.	−1	30	+1	65	−1	12	22	21.81	0.19
4.	+1	80	+1	65	−1	12	27	26.81	0.19
5.	+1	80	−1	25	+1	36	25	23.81	1.19
6.	−1	30	+1	65	+1	36	20	20.56	0.56
7.	−1	30	−1	25	+1	36	25	23.81	1.19
8.	−1	30	−1	25	−1	12	22	22.06	0.06
9.	−2	5	0	45	0	24	21	20.68	0.32
10.	0	55	−2	5	0	24	21	22.43	1.43
11.	0	55	0	45	−2	0	21	21.43	0.43
12.	+2	105	0	45	0	24	24	25.68	1.68
13.	0	55	+2	85	0	24	23	22.93	0.07
14.	0	55	0	45	+2	48	20	20.93	0.93
15.	0	55	0	45	0	24	26	26.22	0.22
16.	0	55	0	45	0	24	26	26.22	0.22
17.	0	55	0	45	0	24	26	26.22	0.22
18.	0	55	0	45	0	24	26	26.22	0.22
19.	0	55	0	45	0	24	26	26.22	0.22
20.	0	55	0	45	0	24	26	26.22	0.22

Note: X_1_ = Concentration of ZnO Nanoparticles; X_2_ = Concentration of Ampicillin/Sulbactam (Ams) antibiotic; X_3_ = Conjugation time; ZOI: Zone of inhibition in mm; Concentration units of ZnO NP and Ams were in µg/mL, and conjugation time was in hour.

**Table 5:** Estimated regression results of different variables.

Effect	SS	MS	F	*p*-Value
“Var1”	4.1329	4.1329	2.6980	0.13150
“Var1^2”	14.5746	14.5746	9.5146	0.01155
“Var2”	5.9065	5.9065	3.8558	0.07796
“Var2^2”	19.7532	19.7532	12.895	0.00492
“Var3”	26.4558	26.4558	17.270	0.00196
“Var3^2”	40.0032	40.0032	26.114	0.00045
“Var1”*“Var2”	8.0000	8.0000	5.2225	0.04537
“Var1”*“Var3”	0.5000	0.5000	0.3264	0.58039
“Var2”*“Var3”	4.5000	4.5000	2.9376	0.11730

Note: Var1 = Concentration of ZnO Nanoparticles; Var2 = Concentration of Ampicillin/Sulbactam (Ams) antibiotic; Var3 = Conjugation time; SS: Sum of square; MS: Mean square; *p*-values less than 0.05 are significant. “*” is the interaction between 2 different variables, whereas “^” is the square term.

**Table 6:** Analysis of variance (ANOVA) for the quadratic model.

Source	SS	DF	MS	F- Value	Prob (p)
Whole model	92.48	9	10.27	6.70	0.0031
Residual	15.31	10	1.53		

*Note:* SS: Sum of squares; DF: degree of freedom; MS: mean square; R = 0.926230; R^2^ = 0.857902; R^2^ (adj) = 0.73001.
